# Editorial: The Comparative Psychology of Intelligence: Macphail Revisited

**DOI:** 10.3389/fpsyg.2021.648782

**Published:** 2021-02-10

**Authors:** Michael Colombo, Damian Scarf, Tom Zentall

**Affiliations:** ^1^Department of Psychology, University of Otago, Dunedin, New Zealand; ^2^Department of Psychology, University of Kentucky, Lexington, KY, United States

**Keywords:** Macphail, null hypothesis, comparative cognition, intelligence, species differences

In a series of papers in the 80s, Macphail (1982, 1985, 1987) put forth evidence in support of the Null Hypothesis for differences in intelligence across non-human species, stating in his 1985 paper that there are “no differences, either qualitative or quantitative, among vertebrates” (p. 46). He further claimed that association formation dominates intelligent behavior, that any differences in intelligence between species could be accounted for by the differential effects of contextual variables, that learning mechanisms are of general applicability and did not evolve as species-specific specializations, and that human intelligence differs from that of other animals in that only humans possess a species-specific language device.

The peer commentaries on Macphail's (1987) *Behavioral and Brain Sciences* paper were generally negative. The most scathing comment was from Goldman-Rakic and Preuss (1987) who suggested that “Macphail's ‘null hypothesis’ is merely the epitaph on the head stone of comparative cognition” (p. 667). Surprisingly, despite the negative tone, Macphail (1987) ended his response to the commentaries on an uplifting note, stating that “For my part, I remain an optimist, and prefer to see the failure to demonstrate differences as evidence not that our scientific procedures are weak but that the animal mind is not what we expected it to be. And after all, did we *really* expect that it would be?” (p. 688). Based on the growth of comparative cognition in the more than three decades since Macphail's (1987) paper, and the papers included in this Research Topic, it is clear Macphail was right to be optimistic. As Pepperberg notes, Macphail (1987) should be given credit for “…instigating a variety of controversies, stimulating the wide-ranging discussions, and generating the types of challenges that have led to many new avenues of research” (p. 10).

At the time, many of the remarkable abilities of non-human animals were unknown to Macphail. Abilities such as episodic memory, theory of mind, orthographic processing, planning for the future, fast mapping, and numerical competence, to name but a few, were yet to have their time in the limelight. With the wealth of comparative data collected over the past 30 years, we thought it was timely to review the status of Macphail's Null Hypothesis, and gauge how the current generation of comparative psychologists approach the inherent challenge that Macphail put forward.

The manuscripts we received ranged from empirical to theoretical, and covered research on a variety of different animals such as pigeons, fish, rats, humans, parrots, eels, crows, monkeys, marine mammals, and spiders. All the papers addressed Macphail's main claim that there are no qualitative or quantitative differences in intelligence across species. A subset of papers addressed Macphail's other claims that (1) contextual variables can explain all of the observed differences between species, (2) associative processes account for all non-human intelligence, and (3) the uniqueness of human intelligence is due to a species-specific language acquisition device.

## Qualitative and Quantitative Differences

Issues of the definition of intelligence aside, virtually all of the manuscripts supported Macphail's view that there are no qualitative differences in intelligence across animals. Whether it is orthographic processing in pigeons (Scarf and Colombo) numerical competence in pigeons, crows, and fish (Scarf and Colombo; Nieder; Petrazzini et al.), counterintuitive features of skill learning in rats (Reid and Swafford), planning in humans (Martin-Ordas), syntactic abilities in parrots (Pepperberg), spatial learning in eels (Watanabe), working memory in crows (Hahn and Rose), the irrational choices of pigeons and humans (Stagner et al.; Zentall), equivalence relations (Colombo and Scarf; Zentall), a variety of other abilities in marine mammals (Bauer et al.) and birds (Bastos and Taylor; Zentall), tool use (Cabrera-Álvarez and Clayton), predatory strategies in spiders (Cross et al.), or complex sequential behavior in rats, mice, pigeons, and humans (Fountain et al.), not to mention a host of other sophisticated behaviors alluded to in many of these papers that have been conducted over the past three and a half decades, all vertebrates seem capable of displaying behaviors that were once considered the domain of only humans or, at most, non-human primates. On the issue of qualitative differences, what might easily be considered the core thrust of the Null Hypothesis, Macphail was nothing short of prescient.

While there was consensus regarding the absence of qualitative differences across species, the majority of the papers were in favor of rejecting Macphail's Null Hypothesis with respect to no quantitative differences across species. The issue of quantitative differences is difficult and depends, of course, on how one chooses to define “quantitative.” Macphail (1985) defined a quantitative difference as “…one species used a mechanism or mechanisms common to both species more efficiently than the other, and this might be reflected in a faster rate of solution or better asymptotic performance…” (p. 38). If defined by asymptotic performance, the fact that the performance of animals on tasks of orthographic processing and numerical competence (Scarf and Colombo), syntactic abilities (Pepperberg), and tool use (Cabrera-Álvarez and Clayton) are comparable between distantly related species (namely birds and primates) lends supports to Macphail's view that there may not even be quantitative differences across species. Cross et al. take this one step further by suggesting that there are even comparable predatory strategies between vertebrate species and spiders (i.e., a non-vertebrate species). That said, most of the papers in the Research Topic accepted that quantitative differences do exist between species, especially if one takes Macphail's definition of “quantitative” as measured by faster rates of solution, or more efficient use of an ability, a vague term that might better be cast as greater flexibility in the use of an ability.

## Role of Contextual Variables

Most of the papers also agree that contextual variables can explain many of the observed differences in abilities across species. The case for the importance of contextual variables was made most strongly by Colombo and Scarf who showed that with respect to a number of tasks, when contextual variables are properly accounted for, qualitative differences that have been observed across different animals vanish. Schubiger et al. presented an enormous list of potential contextual variables, both subject-related and task-related, supporting the possibility that once considered, evidence of even quantitative differences may vanish as well.

## The Role of Associations and The Uniqueness of Human Intelligence

Although few, some papers also tackled Macphail's (1987) claims that association formation underlies intelligence and the uniqueness of human language. With respect to association formation, there were those that supported the idea that association processes underlie cognitive behavior (Scarf and Colombo; Colombo and Scarf), and those that disagreed that cognitive behavior is guided by associative processes (Bastos and Taylor). There seems little doubt that associative processes are universal. Whether Macphail was correct when he stated that association formation dominates intelligent behavior hinges, as so many commentaries in his 1987 paper raised, on how one defines “intelligence.” Bauer et al. were correct when they stated that “In many ways, ‘intelligence’ seems to be a folk psychology term that maps poorly on natural psychological and biological processes, and therefore, lends itself to a wide range of often-inconsistent interpretations” (p 14). Whether behaviors extend beyond associative process is a complex topic. In his typically prescient manner, Macphail (1987) forestalled this issue when he stated that “The problem is that we do not understand what processes underlie…complex behavior” (p. 683).

On the topic of language, countering Macphail's (1987) claim of a species-specific language acquisition device that sets humans apart from other animals, Corballis elegantly highlights the fact that language is now recognized as an amalgam of several abilities (e.g., mental time travel, theory of mind, etc.), many which are present to varying degrees in non-human animals. Further, according to Corballis, it is unlikely that the hierarchical and generative aspects of thought are even unique to humans. Petrazzini et al. echoed similar sentiments in their paper. Thus, if anything, it would seem that Macphail erred on the side of caution with his Null Hypothesis, as it appears that even language may not be a dividing line between humans and non-humans.

## Theoretical Issues

A number of papers also addressed a variety of theoretical issues around the notion of a Null Hypothesis. Petrazzini et al. called into question how we assess the presence vs. absence of differences between species. Traditionally, in keeping with its namesake, Macphail's Null Hypothesis is assessed by rejecting or failing to reject the null hypothesis based on a *p*-value. Petrazzini et al. argued for finer comparative methodologies such as a Bayesian approach, which would evaluate the relative strength of two competing hypotheses. Similarly, Bastos and Taylor also suggested a Bayesian framework to distinguish between support for the Null Hypothesis and a lack of statistical power. Taking this one step further, even when paired with null-hypothesis testing, Bayes factor could be made mandatory for comparative papers, allowing a measure of confidence in any null findings.

Finally, Hahn and Rose argue that working memory is a critical component of cognitive abilities, and that a better way to compare species is to use their working memory capacity and retention limits as a proxy for their cognitive abilities. “Differences and similarities in WM (e.g., in its capacity) may offer insights into why some animals may be (un-)able (*sic*) to display certain cognitive behaviors. Macphail's null-hypothesis can thus be investigated in the light of potentially qualitative, and quantitative differences of a fundamental trait of cognition” (p. 2).

## Conclusions

In light of the results of research over the past 30 years, Macphail's hypothesis that all vertebrates have similar cognitive capacities may not be as implausible as it may have at appeared at the time. In order to conclude that there are qualitative or quantitative differences among species, however, one must first eliminate important differences in contextual variables concerned with perception, motor skills, and motivation. If nothing else, Macphail's proposition has served as encouragement and a valuable challenge to comparative researchers to conduct well designed tests of the abilities of a large number of animal species.

Fountain et al. are correct when they say that “…Macphail's claim continues to challenge all empiricists and theorists to consider the power of even simple neural systems to account for animals' ability to encode simplicity in terms of neural representation from the complexity of the surrounding environmental milieu” (p. 3). With respect to the other issues that Macphail raised, such as whether species differ quantitatively and whether association formation dominates intelligent behavior, Macphail (1987) provided a roadmap as to how the field of comparative cognition can advance our understanding of the human mind by stating that “I express the hope that workers seeking to disprove the null hypothesis will attempt to devise novel tasks for comparative work—tasks which associative devices could not solve” (p. 683).

There is much that the field of comparative cognition owes to Macphail. Corballis stated it perfectly when he said that “Macphail's writing set up the challenge, and attempting to answer it can only advance our knowledge of how animals think, and where humans fit into the overall scheme of things” (p. 8).

## Author Contributions

MC wrote the first draft. DS and TZ reviewed and edited the draft. All authors approved the work for publication.

## Conflict of Interest

The authors declare that the research was conducted in the absence of any commercial or financial relationships that could be construed as a potential conflict of interest.

## References

[B1] Goldman-Rakic P. S. Preuss T. M. (1987). Wither comparative psychology? Behav. Brain Sci. 10, 666–667.

[B2] Macphail E. (1982). Brain and Intelligence in Vertebrates. Clarendon Press.

[B3] Macphail E. M. (1985). Vertebrate intelligence: the null hypothesis. Philos. Trans.R. Soc. Lond. Ser. B Biol. Sci. 308, 37–51.

[B4] Macphail E. M. (1987). The comparative psychology of intelligence. Behav. Brain Sci. 10, 645–695.

